# Advancing risk assessment: mechanistic dose–response modelling of *Listeria monocytogenes* infection in human populations

**DOI:** 10.1098/rsos.180343

**Published:** 2018-08-01

**Authors:** Ashrafur Rahman, Daniel Munther, Aamir Fazil, Ben Smith, Jianhong Wu

**Affiliations:** 1 Laboratory for Industrial and Applied Mathematics, Centre for Disease Modelling, Department of Mathematics and Statistics, York University, Toronto, Ontario, Canada M3J 1P3; 2 Department of Mathematics, Cleveland State University, Cleveland, OH 44115, USA; 3 National Microbiology Laboratory, Public Health Agency of Canada, Guelph, Ontario, Canada N1G 5B2

**Keywords:** *Listeria monocytogenes*, dose–response, mechanistic model, human population, comparative study

## Abstract

The utility of characterizing the effects of strain variation and individual/subgroup susceptibility on dose–response outcomes has motivated the search for new approaches beyond the popular use of the exponential dose–response model for listeriosis. While descriptive models can account for such variation, they have limited power to extrapolate beyond the details of particular outbreaks. By contrast, this study exhibits dose–response relationships from a mechanistic basis, quantifying key biological factors involved in pathogen–host dynamics. An efficient computational algorithm and geometric interpretation of the infection pathway are developed to connect dose–response relationships with the underlying bistable dynamics of the model. Relying on *in vitro* experiments as well as outbreak data, we estimate plausible parameters for the human context. Despite the presence of uncertainty in such parameters, sensitivity analysis reveals that the host response is most influenced by the pathogen–immune system interaction. In particular, we show how variation in this interaction across a subgroup of the population dictates the shape of dose–response curves. Finally, in terms of future experimentation, our model results provide guidelines and highlight vital aspects of the interplay between immune cells and particular strains of *Listeria monocytogenes* that should be examined.

## 1. Introduction

As a foodborne disease, human listeriosis caused by *Listeria monocytogenes* is relatively rare. While outbreaks due to *L. monocytogenes* are much less common as compared with those linked to *Escherichia coli* and *Salmonella*, their occurrence is associated with a high case fatality rate (20–30%) [1,2]. Pregnant women, immunocompromised individuals and the elderly are particularly susceptible to this potentially deadly disease [3,4]. Considering medical costs, productivity loss and valuation of premature mortality, Hoffmann *et al.* estimated that the annual cost of illness in the USA due to *L. monocytogenes* is $2.6 billion [5].

Given the severity of the consequences within specific subgroups of the population and the continued socio-economic burden, much research has been devoted to elucidating the infection processes of *L. monocytogenes* [4]. For instance, drawing from ‘humanized’ transgenic murine models as well as guinea pigs and rabbits, scientific studies have explored the intracellular transmission mechanisms of this pathogen as well as its ability to cross the intestinal wall [3,4,6]. More than 50 surface proteins have been shown to be involved with host cell invasion and cell-to-cell movement [3]. In terms of cell-to-cell contact, *L. monocytogenes* expresses surface proteins internalin, intA and intB, that interact with the host's receptors E-cadherin and Met [7,8]. Pore-forming gene listeriolysisn O (LLO) aids bacterial escape from the vacuole of a cell and surface protein ActA promotes intra- and inter-cellular movement. Other proteins such as Clathrin, Lecitinase, Septin and Tuba facilitate the invasion process and help *L. monocytogenes* to avoid immune surveillance [4]. After crossing the intestinal wall, the pathogen most likely disseminates from the mesenteric lymph nodes and blood vessels to the spleen and liver, potentially reaching the brain and placenta [3,4,6,9].

In line with animal-based studies focused on the pathophysiology of listeriosis, the infection potential of *L. monocytogenes* has been shown to be dose-dependent [6,9,10]. An inoculation with 1×10^11^ CFU of the pathogen indicates a 100% fatality rate in mice, whereas a dose of 1×10^10^ CFU results in 100% survival [6]. Van Stelten *et al.* show that both the number of guinea pigs and the proportion of organs infected increase with higher inoculation doses [11]. For doses 1×10^6^, 1×10^7^ and 1×10^10^ CFU of *L. monocytogenes*, the respective infection rates were 0%, 50% and 100%. Similar experiments with monkeys [10], mice [12–14] and zebrafish [15] demonstrate that casualty, systemic infection and pregnancy disorders are strongly linked with the magnitude of inoculation doses.

Logically building from the understanding that listeriosis is dose-dependent, the quantification of dose–response relationships has typically been developed via statistical models supported by animal studies and human outbreak data (e.g. [2,6,16–19] and references therein). While this research is valuable for correlating dose and response in particular circumstances (for instance, after an outbreak has occurred), without a mechanistic understanding of how biological factors, relative to dose, dictate individual host infections, such correlative descriptions have limited predictive capacity [20,21]. To bridge the gap between exposure and infection, a mechanistic description is crucial [3,16].

A recent approach in this regard concerns the Key Events Dose–Response Framework (KEDRF) [20]. The KEDRF attempts to characterize the respective cascade of steps and transfer probabilities involved, for instance, within the infection pathway of an invasive pathogen such as *L. monocytogenes*. Current dose–response models for such pathogens typically depend on two key assumptions: (i) single hit (i.e. a single ingested pathogen has the potential to cause infection) and (ii) independent action (i.e. the probability of infection caused by any particular pathogen is independent of the number ingested) [20]. While these assumptions are widely accepted, they have a significant impact on the shape of the dose–response curve at low doses. For instance, a study by Holcomb *et al.* compared six dose–response models, illustrating vast differences in their respective responses when extrapolating from high to low doses [22]. Because of these limitations and the difficulty of obtaining useful data at low doses, Buchanan *et al.* suggest that ‘the best strategy for refining dose–response assessment for foodborne pathogens is to advance the understanding of the underlying biology, and by doing so, refine the assumptions that underline predictive models’ [20]. This is where mechanistic modelling can play a vital role. That is, by quantifying the fundamental biology involved in the key steps of the infection pathway, and using these to generate dose–response relationships, predictions of infection risk can be greatly improved [20,21].

Currently, there are a limited number of studies that incorporate mechanistic modelling to study the dose–response relationship. Pujol *et al.* developed a dynamic model to demonstrate how immune system effects produce dose–responses relative to the timing of pathogen dose [23]. In particular, they showed that models which focus only on single exposure could overestimate the infection risk. While their model highlights general aspects of immune response, they do not include the effect of gastric pressure in mitigating the probability of infection. In the context of the classical SIR model, Brouwer *et al.* demonstrated that the impact on pathogen transmission with regard to the choice of dose–response functional form can vary markedly [24]. Furthermore, other studies such as [25,26] have explored mechanistic approaches to draw conclusions from low dose exposure in the context of radiation/cancer onset and as well as norovirus infection.

In terms of *L. monocytogenes*, there is a paucity of research in which the quantification of infection risk is based on detailed mathematical descriptions of host–pathogen interactions [27]. As a primary step in addressing this critical need, we built a mechanistic model that describes the gastrointestinal pathway of *L. monocytogenes* survival within a host [28]. Applying the model to guinea pigs, we quantifiably characterized the dose–response relationship in terms of parameters that represent the innate immune response and stress of the gastrointestinal environment. This work [28] represents important progress towards developing a new paradigm, as Haas terms it ‘models beyond Generation 3’, in which microbial dose–response modelling incorporates mechanistic details of *in vivo* physiological processes [16].

In the interest of public health, however, such a model should reflect the kinetics and progression of a bacterial infection in humans. One of the limitations of the model in [28] is that its parameter values and functional forms are determined using guinea pig data. The goal of the present paper is twofold: (i) to address this issue by using *in vitro* studies that simulate human physiology, modified mathematical forms and human listeriosis outbreak data to develop a more relevant mechanistic model and (ii) in the context of the Finland butter outbreak [18], to compare the dose–response generated from the exponential form (used by FAO/WHO) with the dose–response relationship arising from our new model. To that end, this paper is organized as follows.

Section 2 describes the gastrointestinal pathway of *L. monocytogenes* in human hosts and the potential host barriers against harmful pathogens. A mechanistic dose–response model is developed in §3. This section includes complete model analysis as well as the estimation of key parameter ranges as applied to human infection. Section 3 also describes the biological implications and computational aspects of the model. In order to address uncertainties in the model parameters, and to further clarify conditions under which specific parameters play a dominant role in determining infection outcomes, we perform a sensitivity analysis in §4. In §5, we use human outbreak data to help elucidate the ranges of those parameters that govern immune response. Lastly, in §6, we discuss the utility of the model as a new microbial risk assessment tool, highlighting the need for experimentation to simulate/quantify specific immune response features in the human small intestine as well as the effect of different food matrices on the survivability of *L. monocytogenes* in the stomach.

## 2. Gastrointestinal barriers for *Listeria monocytogenes*

*Listeria monocytogenes* is a foodborne pathogen. To survive in a human host, we consider the following challenges the pathogen must overcome along the pathway from ingestion to gastrointestinal colonization and potential infection.

### 2.1. Gastric juice and low pH

After ingestion, *L. monocytogenes* travels along with food particles through the oesophagus and reaches the stomach within a few seconds (in this study, we disregard the effect of saliva in the mouth on the bacterial population). Once in the stomach, enzymes and acid are secreted to sanitize and break food into digestible molecules. Among these secretions, stomach acid (HCl) is significant for a number of reasons. It initiates protein digestion by activating pepsinogen that secretes from the gastric gland. It also enhances the absorption of minerals, calcium and iron [29]. In addition, stomach acid plays a crucial role in clearing food pathogens from the stomach before they move down to the small intestine. Numerous *in vitro* and *in vivo* experimental results indicate that low pH levels are detrimental for bacterial survival [30–32].

For instance, scientific experiments with rats [33], mice [34] and guinea pigs [6] showed that a significant portion of inoculated *L. monocytogenes* are killed following the first hours of ingestion. These data indicate that stomach fluid is a major deterrent for the survival and growth of *L. monocytogenes*. In fact, stomach fluid reduces the number of *L. monocytogenes* by more than 99% [9,30]. If the bacteria can manage to survive the stomach fluid and reach the small intestine, where the pH level is relatively neutral (6.4–7.4) [35], they may be able to replicate and grow.

### 2.2. Commensal bacteria in the small intestine

Upon surviving the gastric environment, *L. monocytogenes* passes to the small intestine. While the small intestine is a more favourable environment for bacterial persistence as compared to the stomach, the bacteria still need to overcome several obstacles to colonize. The small intestine is a niche for more than 500 species of commensal microbiota [36,37]. The commensal bacteria are beneficial for the host, but fight against incoming pathogens through different mechanisms. A recent study shows that commensal microbes provides the first line of defence against *L. monocytogenes* in the small intestine [38]. By consuming unused nutrients, commensal microbiota compete with other pathogens for resources and inhibit their growth. In addition, by releasing microbial metabolites, acetate and antimicrobial effector molecules called bacteriocins, resident microbiota can effectively inhibit colonization of pathogenic bacteria [37,39]. Microbiota also induce innate and adaptive immunity by releasing microbial patterns, lipopolysaccharides and peptidoglycan, which can be sensed by host epithelial cells. Upon sensing pathogens, goblet cells release gel-forming mucins and epithelial cells release defensins [37]. These proteins help kill bacteria and defend the host from any chemical and physical injuries due to pathogenesis [37,40].

### 2.3. Immune cells

The innate immune system is the first line of defence against pathogen colonization and is primarily responsible for clearing pathogens from the host. Multiple components of the immune system may be activated simultaneously to eliminate pathogens. Dendritic cells (DCs) and macrophages are two important components of the innate immune system and are effective, in particular, against *L. monocytogenes*. DCs sample luminal contents by stretching their long dendrites through tight junctions of epithelial cells. Macrophages destroy pathogens by uptaking and degrading them, releasing inflammatory mediators and inducing adaptive immune response [37,41,42]. These immune cells have the ability to recognize microbial patterns through pattern recognition receptors and toll-like receptors [43]. In addition, the role of bactericidal lectin RegIIIγ secreted by intestinal paneth cells is found to be remarkable. MyD88-mediated signalling pathway induces a high level of RegIIIγ that clears the *L. monocytogenes* from the small intestine rapidly [30,44]. The innate immune system in essence regulates the whole immune response against the invading pathogens and attempts to prevent them from spreading beyond the intestine and causing systemic infection.

When an infection persists, the adaptive immune system becomes activated. CD8^+^ T cells proliferate rapidly and clear the infection [45]. Plasma cells mediated by DCs release *I*_*g*_*A* in the lamina propria [36,37]. *I*_*g*_*A* can reach the small intestine and attach to bacteria to inactivate them [37]. Both T cells and B cells retain memories which can promptly clear a possible secondary infection [36,45].

In addition to microbial resistance and immune pressure, the liver secrets a significant amount of bile into the intestinal tract which exerts stress on pathogens [46,47]. Bile can affect phospholipids and proteins of cell membrane and structure, and cause dissociation of the cell membrane [48]. It also induces DNA damage in bacterial cells [47]. However, the bile resistance of *L. monocytogenes* in the small intestine has also been reported [49,50].

## 3. Mechanistic dose–response model

### 3.1. Population dynamics of *Listeria monocytogenes* in the human gut pathway

In this section, we describe the mechanistic dose–response model which is an extension of a previous model [28] developed in the context of animal data. Food particles are the natural vehicles of *L. monocytogenes* for the in-host exposure. In this study, we will consider the initial pathway for *L. monocytogenes* from mouth to small intestine in the human context. Considering the environment along the gut pathway as described in the previous section, we view the population dynamics of *L. monocytogenes* in the stomach and small intestine separately. In particular, we assume that (1) bacteria do not grow in the stomach due to the presence of high acidity, but are killed, decaying exponentially. (2) Since food passes continuously from the stomach right after consumption [51,52], we assume that while stomach acid kills the bacteria, bacteria are also being discharged with food particles continuously to the small intestine from the stomach at a rate of *σ* per hour. (3) The growth of the bacteria is limited by commensals, immune pressure and the intestinal environment. That is, bacteria cannot grow beyond the carrying capacity of the small intestine. (4) In the small intestine, *L. monocytogenes* can reproduce at a rate of *r* per hour and may be killed by the immune cells and intestinal fluid (e.g. bile) at a rate of *β* per hour; see [28] for details.

Following assumptions (1)–(4) above, the dynamics of *L. monocytogenes* in the gut is described by the following ordinary differential equation model:

3.1 $$\begin{array}{rl} L_{g}^{\prime } & =-\delta L_{g}-\sigma L_{g}\\ \;\text{and}\;L_{i}^{\prime } & =\sigma L_{g}+rL_{i}\left(1-\frac{L_{i}}{K}\right)-\frac{\beta L_{i}}{1+\alpha L_{i}}, \end{array}\}$$

where *L*_*g*_ (CFU) denotes the population of *L. monocytogenes* in the stomach at time *t* (in hours) and *δ*>0 (h^−1^) represents the kill rate of the bacteria due to high acidity in the gastric environment. *L*_*i*_ (CFU) represents the population of *L. monocytogenes* in the small intestine and we assume the population dynamics there follow a logistic growth function with an intrinsic growth rate *r* and carrying capacity *K*. Owing to host defence, *L. monocytogenes* is killed at the rate of *β* with a saturated killing mechanism involving the parameter *α* [53,54]. Briefly, *α* quantifies the potential of the pathogen to escape the host's defence. If the number of bacteria (*L*_*i*_) or value of *α* is large, then the bacteria can outcompete the host response. The details regarding the mechanism of saturated killing and rationality behind the mathematical form used are discussed in [28]. A summary of the parameters and variables of model (3.1) is given in table 1.

**Table 1.** Description of the variables and parameters of model (3.1).

variables and parameters	description
*L*_*g*_	population of *L. monocytogenes* in the stomach
*L*_*i*_	population of *L. monocytogenes* in the small intestine
*δ*	killing rate of *L. monocytogenes* in the stomach
*r*	growth rate of *L. monocytogenes* in the small intestine
*K*	carrying capacity of *L. monocytogenes* in the small intestine
*σ*	dispersal rate of *L. monocytogenes* from stomach to the small intestine
*β*	killing rate of *L. monocytogenes* in the small intestine
*α*	defence potential of *L. monocytogenes* against the host in the small intestine

### 3.2. Model (3.1) analysis and implications

#### 3.2.1. Classification of mathematical dynamics

Model (3.1) is two-dimensional and has relatively simple dynamics. It is well posed in the sense that solutions of the model are uniquely defined and positive for any positive initial values. The model has three possible steady states: *L*_0_(0,*L*_0_),L_*_(0,L_*_) and *L*_+_(0,*L*_+_), where

$$\begin{array}{rl} L_{0} & =0,\;\\ L_{*} & =\frac{rK\alpha -r-\sqrt{r^{2}K^{2}\alpha^{2}+2r^{2}K\alpha +r^{2}-4r\alpha \beta K}}{2r\alpha }\\ \;\mathrm{and}\;L_{+} & =\frac{rK\alpha -r+\sqrt{r^{2}K^{2}\alpha^{2}+2r^{2}K\alpha +r^{2}-4r\alpha \beta K}}{2r\alpha }. \end{array}$$

Notice that the first component of each of the steady states is zero and thus we label each steady state by its second component.

To describe the dynamics of model (3.1) completely, we adopt a geometric approach, illustrating solution behaviour relative to three mutual exclusive cases that are characterized by the existence of steady states.

Using the fact that the nullclines of *L*_*g*_ and *L*_*i*_ are given by

**Figure 1.**
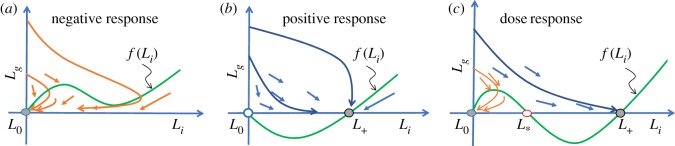
Illustration of steady states and vector field governing the solutions of model (3.1). The *L*_*i*_-nullcline is represented by *f*(*L*_*i*_) and the *L*_*i*_-axis represents the *L*_*g*_-nullcline. The vector field demonstrates the possible course of direction of the model solutions as they start from an initial value (dose) on the *L*_*g*_-axis. The filled circles represent stable steady states while open circles represent unstable steady state. Three distinct scenarios arise from threshold conditions. (*a*) *r*< 4*αKβ*/(1+*αK*)^2^: solutions always approach the 0 steady state (*L*_0_), indicating bacterial extinction independent of initial conditions; (*b*) *β*<*r*: solutions always approach the positive steady state (*L*_+_), showing bacterial persistence independent of initial values; (*c*) 4*αKβ*/(1+*αK*)^2^<*r*<*β*: solutions can approach either *L*_0_ or *L*_+_ depending on the initial value (dose), creating a scenario that can generate dose–responses.

— *L*_*g*_-nullcline: *L*_*g*_=0
— *L*_*i*_-nullcline: *L*_*g*_=(1/*σ*)(*βL*_*i*_/(1+*αL*_*i*_)−*rL*_*i*_(1−*L*_*i*_/*K*))=*f*(*L*_*i*_)

and the fact that the derivative of *L*_*g*_ is always negative, we have the following cases:

Case I (only *L*_0_ exists): In this case, *L*_0_ is globally stable and the dynamics are relatively simple. The trajectories beginning from the *L*_*g*_-axis will move to the southeast direction until they hit the *L*_*i*_–nullcline (*f*), then will follow the southwest direction until reaching *L*_0_ as can be seen in figure 1*a*. This case exists when *r*<4*αKβ*/(1+*αK*^2^) (<*β*).

Case II (*L*_0_ and *L*_+_ exist): In this case, *L*_+_ is globally stable and the dynamics of the model are also simple. The trajectories from the *L*_*g*_-axis will always approach *L*_+_ following the southeast direction (figure 1*b*). This situation occurs when *β*<*r*, and like case I, the model behaviour does not depend on the initial conditions.

Case III (all three steady states exist): In this case *L*_0_ and *L*_+_ are locally stable and L_*_ is unstable, there is a boundary (separatrix) between the basins of *L*_0_ and *L*_+_ originates from L_*_ (figure 1*c*). This case exists when 4*αKβ*/(1+*αK*)^2^<*r*<*β*. We should note that *α* must be positive for the existence of case III.

#### 3.2.2. Biological implications and dose–response

The governing perspective connecting model (3.1) with infection outcomes in humans is this: selecting a person from a given population and a strain of *L. monocytogenes* translates to assigning specific values to each parameter in the model, selecting an ingested dose and running the model. The respective parameter values corresponding to the specific host and pathogen strain characteristics in question fall into one of the three cases indicated above. Notice that *βK*/(1+*αK*) quantifies the maximum killing rate of the pathogen via immune cells, and 4*α*/(1+*αK*)<1 (for positive *α*) can be thought of as a fractional scaling related to the virulence of the pathogen in question. In the light of this, case I represents a strong host defence coupled with a strain having minimal growth potential in the small intestine. In terms of bacterial dynamics, case I implies that no matter the initial dose, the bacteria will not be able to colonize the gut, but will be driven to extinction. Like case I, case II does not depend on initial conditions, but given an arbitrary initial dose, the bacteria will always be able to colonize the gut, leading to infection. Thus, case II represents a strain with a rapid growth potential in conjunction with poor host defence.

**Figure 2.**
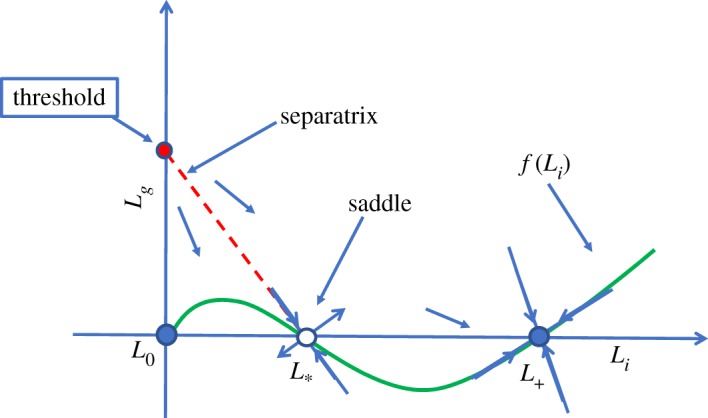
Threshold approximation. The extension of the eigenvector, emanating from L_*_, to the *L*_*g*_-axis approximates the separatrix of the two basins.

In terms of the aforementioned parameter conditions, case III represents a wide range of host immune strengths and pathogen strain characteristics. In addition, case III differs dramatically from the other two as the solution behaviour depends on initial conditions. If the dose is large enough, then model (3.1) predicts that the bacteria will colonize the gut, whereas, for smaller doses, colonization is not possible. Mathematically speaking, this dose threshold depends on where the separatrix intersects the *L*_*g*_ axis (figure 2).

In order to determine the dose–response relationship predicted via model (3.1), we run it with its given parameter and initial (dose) values, observing whether the solution approaches *L*+ or not. To find the probability of positive response, we run the model *n* times with the distribution of model parameters under a fixed dose and count the number of positive responses (*c*) out of *n* runs. The probability of infection is calculated as *c*/*n* for that dose. Notice that while the implication that colonization implies host infection may not always be true, for the rest of the paper, we will assume that it holds. Henceforth, we will refer to colonization and infection interchangeably unless otherwise indicated. For a more detailed treatment of this notion and related ramifications, see §6.

#### 3.2.3. Generation of dose–response: model (3.1) versus traditional approaches

In order to illustrate the fundamental difference in approach for model (3.1) compared with traditional dose–response, we consider single hit models. Single hit models describe a dose–response in essentially two steps: (1) the probability of being exposed to *j* pathogens given a mean dose *d* and (2) the probability that *n* of *j* pathogens survive host defences and cause infection. The probability of infection given a dose *d* is effectively determined by the discrete convolution of the probabilities given in (1) and (2) [16]. Furthermore, these models are based on the assumption that a single pathogen may survive and lead to host infection, in contrast to ‘threshold models’ or ‘Generation 0’ models [16] that are built from the supposition of an ‘infectious dose’, above which infection is possible and below which infection cannot occur. In terms of representing biological reality, single hit models are considered more plausible.

For the classic exponential model, for instance, the probability of infection given a dose *d* is given by

3.2 P(d)=1−exp⁡(−Rd),

where each pathogen has an independent and equal probability *R* to survive and cause infection [16]. Note that the exponential model depends on the perspective that pathogen exposure can be described by a Poisson distribution, determining the probability in (1) above, and given the independence assumption of pathogen survival/infectivity, the resulting binomial distribution provides the probability in (2) above [16]. The crucial notion here is that the assumption of independent identical survival of the pathogens effectively removes variability among respective hosts [16].

In marked contrast to this, model (3.1) generates the dose–response function from the population-level variation of model parameters. In essence, each member of the population has a dose threshold determined by their personal immune status, physiology and the characteristics of the ingested pathogen strain. Mathematically, recall that this threshold, for case III, is determined by the intersection of the separatrix and the *L*_*g*_ axis (figure 2). (Note that for case I, this threshold is effectively infinite, whereas for case II, the threshold is zero.) However, our model is not a typical ‘threshold model’ in that it is still possible that a single organism can cause infection. For instance, if the flow rate from stomach to small intestine *σ* is large enough, a single pathogen can survive ingestion and enter the small intestine. Furthermore, for relatively large *α*, the pathogen's potential of escape from the host's defence in the gut, model (3.1) accounts for the possibility that a single pathogen can initiate infection. For more details concerning the sensitivity of the dose threshold relative to the model parameters, see §4.

#### 3.2.4. Computational cost and analytical tools

In order to predict the dose–response from model (3.1), we need to run/simulate the model with its parameter values. The solution approaches either the *L*_0_ or *L*_+_ state (negative or positive response, respectively; figure 1). To find the probability of infection for a certain dose *d*, the model needs to be run *n* times by randomly selecting host parameters for each run from their respective distributions. Owing to the random sampling of the parameters, the existence of the steady states and the dynamics as well as the outcomes of the model could be different for each run. If there are *c* positive responses out of *n* (i.e. the solution approaches *L*_+_ *c* times out of *n*) runs then the infection probability would be *c*/*n* at dose *d*. The value of *n* depends on the number of samples we take from the parameter space. It also critically depends on how small a probability we are interested in determining. To compute a probability of 1×10^−10^ (as reported in [17]) associated with a certain dose *n* should be more than or equal to 1×10^10^, otherwise, the model could predict 0 probability. This leads to impractically massive calculations on two fronts: (i) generating dose–responses at relatively low doses and (ii) using model (3.1) outputs to help determine parameter ranges against outbreak data.

To help reduce such computational cost, we use a geometric approach to predict positive or negative responses without solving model (3.1) directly. In particular, we seek to determine the threshold on the *L*_*g*_-axis, in terms of model parameters, so that for the initial value (dose) above the threshold the trajectory will approach *L*_+_ and from below the threshold it will approach *L*_0_. Therefore, by comparing a given initial dose to this threshold, we can then predict whether it will lead to a positive or negative response in the host.

Based on our analysis in §3.2.1, the bistable phenomenon we seek only occurs for parameter values in case III. In particular, the initial dose threshold is determined by the intersection of the separatrix with the *L*_*g*_-axis. This boundary curve is part of the stable manifold for the steady state L_*_, ‘connecting’ the *L*_*g*_-axis to the steady state L_*_ (figure 2). While globally defining the stable manifold for L_*_ in terms of the model parameters may not be possible, we use a linear approximation.

The eigenvalues of the linearized system of model (3.1) at L_*_ are given by

$$\begin{array}{rl} \lambda_{1} & =r-\frac{2rL_{*}}{K}-\frac{\beta }{1+\alpha L_{*}}+\frac{\alpha \beta L_{*}}{{\left(1+\alpha L_{*}\right)}^{2}}\\ \;\mathrm{and}\;\lambda_{2} & =-(\delta +\sigma ). \end{array}$$

The eigenvector corresponding to the eigenvalue λ_2_ is given by the equation

3.3 $$L_{g}=m(L_{i}-L_{*}),$$

where

$$m=\left[-(\delta +\sigma )+\frac{rL_{*}}{K}-\frac{\alpha \beta L_{*}}{{\left(1+\alpha L_{*}\right)}^{2}}\right]\frac{1}{\sigma }.$$

An approximation to the desired threshold is obtained when line (3.3) hits the *L*_*g*_-axis and is given by

3.4 $$L_{g}(0)\;=\;-\;mL_{*}.$$

**Figure 3.**
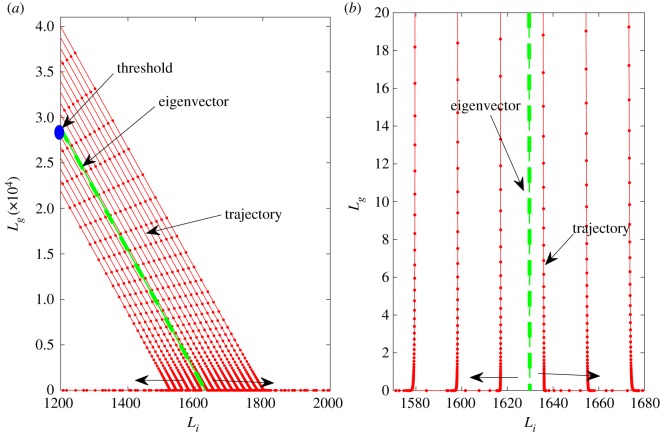
Demonstration of the behaviour of trajectories and significance of threshold (3.4) to determine the destination of trajectories. Trajectories starting above (below) the threshold travel parallel to the eigenvector (3.3), then along the *L*_*i*_-axis, approaching the steady state *L*_+_ (*L*_0_). (*a*) The full view and (*b*) a magnified view near the *L*_*i*_-axis.

The phenomenon is depicted in figure 2 and in the numerical simulation figure 3.

Now we explain how to use this threshold to obtain the dose–response. Instead of solving model (3.1) to find the endpoint of the trajectory, we just compute the threshold −mL_*_ and check if the initial dose is above or below this threshold. For a given sample of parameters, if the initial dose is larger than −mL_*_, we count (predict) the event as a positive response. Thus the dose–response problem is simplified to: given a dose *d*, find

3.5 $$\mathrm{Prob}\{d\;>\;-\;mL_{*}\}.$$

**Figure 4.**
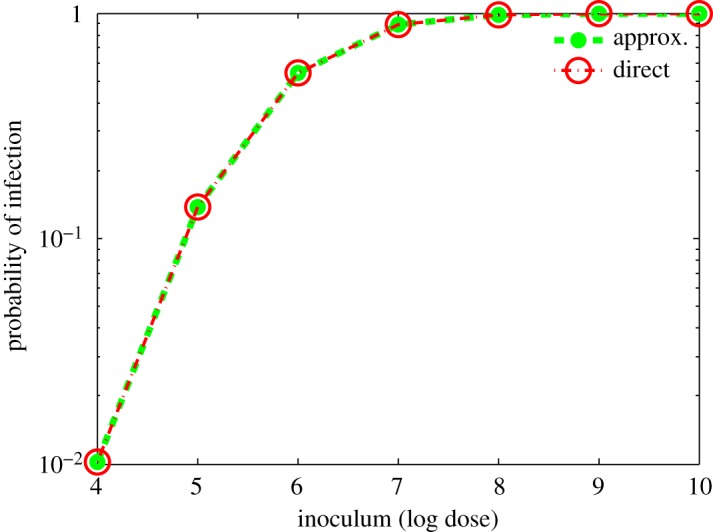
Dose–response curves computed by using threshold approximation and exact solution. The dose–response curves from the two approaches are significantly close, in fact identical. 10^3^ samples are taken from the baseline parameter space (table 2) for this comparison.

This new technique makes the whole computational process significantly faster as it does not require finding long-time solutions of the model. The accuracy of the prediction, however, depends on selecting the model parameters from certain regions of parameter space. In the next section, we determine parameter ranges so that model (3.1) represents the infection pathway in the human context and that using our linear approximation gives essentially the same results as obtained by solving model (3.1). Figures 3 and 4 show the accuracy of the prediction of the threshold approximation relative to the baseline parameters. We also tested the approximation method with other parameter combinations (satisfying criteria for case III), including extreme values, and found similar results (not shown here). This gives us confidence to use the threshold approximation for the rest of the results. In figure 3, the trajectories travel parallel to the essentially linear separatrix and then closely follow the *L*_*i*_-axis as they approach the respective steady states.

**Table 2.** The baseline values of the model parameters.

parameter	description	baseline value (mean ± s.d.)	references
*r*	growth rate	0.863±0.43 h^−1^	[59]
*K*	carrying capacity	10^8±1^ CFU	[10]
*σ*	dispersal rate	0.4832±0.1419 h^−1^	[52]
**β**	killing rate	4.90±0.75 h^−1^	data fit, [18]
*α*	saturating factor	10^−3.35±0.53^ CFU^−1^	data fit, [18]

### 3.3. Parameter estimation

Model (3.1) has six parameters which are linked to the underlying biology: *δ,σ,β* and *K* relate to the individual hosts, while *r* and *α* are specific to bacterial strain as well as particulars with regards to the environment of the host's gut. To compare the dose–response generated from the mechanistic model (3.1) with alternate dose–response models [17] that rely on human epidemiological data, we need appropriate values for these parameters. Recall that the population-level variation in model parameters naturally gives rise to the dose–response function governed by model (3.1). Therefore, the choice of sampling distribution on parameter space becomes important. Owing to the fact there is little known information concerning the details of the distributions of these parameters in the human context, we assume that all model parameters are distributed normally across their respective ranges. Note that in §§4 and 6, we provide more discussion regarding the sensitivity of model (3.1) generated dose–response function to various parameter distributions. In what follows, we determine biologically feasible ranges of each of these parameters in the context of human subjects.

#### 3.3.1. Estimation of bacteria killing rate (*δ*) in the stomach 

The parameter *δ* is associated with bacteria killing in the stomach due to acid. The acidity or pH level of the stomach depends on the emptiness of the stomach. Human stomach pH ranges between 1 and 3, but could go beyond 5 after food consumption [55]. For instance, the fasting stomach pH ranges between 1.3 and 1.7, but rapidly increases to around 5.0 followed by a gradual decrease during the stomach emptying time. Dressman *et al.* [56] fit an exponential model to human stomach pH following food consumption and found

$$\mathrm{pH}(t)\;=\;4.13\;\times \;10^{\;-\;0.3t}.$$

In an *in vitro* experiment, Zhu *et al.* [32] found that *E. coli* bacteria hardly survive at pH 2.5, but they can survive about an hour and half at pH 3.0. However, when pH increases to 4.0 and beyond, the bacteria not only survive for a longer period but also manage to replicate. Similar results were found on the survival of *L. monocytogenes* on Sapote mamey pulp at various pH levels [57]. To estimate *δ* in terms of pH, we fit an exponential model

3.6 $$\delta (pH)\;=\;\delta_{0}\;e^{\delta_{1}\;\mathrm{pH}}$$

to bacterial growth data [32] and found *δ*_0_=260 220 and *δ*_1_=−3.4. The mean value of *δ* over a 2 h period is estimated to be 20.0 h^−1^. After this 2 h period, pH drops to a level at which bacteria can hardly survive.

#### 3.3.2. Estimation of dispersal rate (*σ*)

The parameter *σ* is related to bacteria dispersal from the stomach to the small intestine. Since bacteria pass along with food, it is reasonable to assume that the bacteria dispersal rate is the same as the food dispersal rate. The dispersal of food or stomach emptying time depends on the individual as well as on the food matrix. A liquid can be passed rapidly while solid food takes longer to be processed. An *in vivo* study found that the stomach emptying time is exponentially distributed with a median half-time, *T*_1/2_, for solid and liquid foods, of 127 min and 80.5 min, respectively [51]. A similar study shows that the gastric emptying half-time and per cent gastric retention at 2 h are 68.7 min and 16.3%, respectively [52]. Considering these studies, we estimate the mean and standard deviation (s.d.) of dispersal rate *σ* to be 0.4832 h^−1^ and 0.1419, respectively.

#### 3.3.3. Estimation of growth rate (*r*)

The parameter *r* accounts for the growth or replication rate of *L. monocytogenes*. The estimation of the growth rate of bacteria in the human gut from an *in vivo* experiment is unrealistic. In terms of an animal model, the growth rate of *L. monocytogenes* in the small intestine of a guinea pig was estimated to be 0.23 h^−1^ [28]. Schvartzman *et al.* found a growth rate of 1.20 h^−1^ in cheese medium [58]. A comprehensive review on bacterial growth found that the average growth of *L. monocytogenes* in five different growth media (meat products, cheese, seafood, microbiological media and liquid dairy product) is 0.863 h^−1^ with s.d. of 0.43 that ranges between 0.24 and 1.37 [59]. We consider *r*=0.863 h^−1^ and carry out sensitivity analysis to observe the effect of *r* on the dose–response curve. However, this could be an overestimate as the small intestine is stressful for bacteria [37] as compared to the *in vitro* media [59].

#### 3.3.4. Estimation of carrying capacity (*K*)

We assume that *L. monocytogenes* grows at the rate of *r* in the small intestine, where the pH level is relatively neutral. However, bacteria cannot grow unbounded and are limited by the environmental capacity. The carrying capacity *K* in the small intestine may depend on multiple factors including nutrition, space and location, competition with other organisms and the detrimental effect of chemicals and molecules. While the carrying capacity of *L. monocytogenes* in the human small intestine has not been estimated through *in vivo* experiments (due to the obvious limitations), we estimate a plausible value from various studies. An *in vivo* experiment shows that *L. monocytogenes* can grow in the small intestine of guinea pigs to 3×10^6^ CFU [6]. In a monkey experiment, the maximum number of *L. monocytogenes* in the colon is found to be 1.7×10^7^ CFU [10]. Melton *et al.* found the number of the bacteria in the colon of guinea pigs is around 10^4^ CFU [9]. Since the human gut is much larger than some of these studied animals, we consider the carrying capacity of *L. monocytogenes* ranges from 10^7^ to 10^9^ CFU with mean 1.0×10^8^ CFU. We also carry out a sensitivity analysis for *K* in §4 to observe the effect of *K* on the dose–response curve.

#### 3.3.5. Estimation of killing rate (*β*) and defence potential (*α*)

Because we assume the inactivation of *L. monocytogenes* is governed by Michaelis–Menten kinetics, *βL*_*i*_/(1+*αL*_*i*_) in model (3.1), the parameters *β* and *α* must be determined relative to this mathematical form. The parameter *β* represents the killing rate of bacteria in the small intestine by the host. This is presumably due to the host's macrophages which make up part of its innate immune system. Other immune cells, e.g. dendritic cells and plasma cells, take part in prevention of bacterial infection in the small intestine; however, the role of macrophages in bacterial killing is well documented [37,41]. On the other hand, the parameter *α* can be regarded as the failure of host defence, particularly when the number of bacteria becomes large. In other words, it is the potential of pathogens to evade the host's defence.

To find meaningful ranges for *β* and *α* via data fitting techniques, care must be taken as *α* is sensitive to *L*_*i*_. That is, these parameters should ideally be determined from a single study that considers various initial pathogen doses. For instance, in a study by Noerjasin, listericidal activity by human macrophages cultured from peripheral blood monocytes was determined [60]. While Noerjasin considered both ‘stimulated’ and ‘non-stimulated’ cases, the lack of variation in the initial inoculation dose precludes model fitting to find appropriate *α* and *β* values [60]. Even if Noerjasin had considered a range of initial inoculum levels, data from the study may be of limited use since intestinal macrophages differ somewhat in their defensive activities as compared to blood monocytes or macrophages in other tissues [42].

Owing to limited human data on this front, and the fact that mouse models have been widely used to study the immune response against *L. monocytogenes* because of the genetic and physiological similarities between a mouse and a human [18,45], we consider a study by Higginbotham *et al.* who reported an *in vitro* experiment regarding the killing of *L. monocytogenes* by mouse macrophages [41]. It shows that macrophages can kill a significant portion of *L. monocytogenes*. From this study, we estimate mean values of *β*=0.173 and *α*=6.76×10^−8^. However, since *β* on average is less than the range of values for *r* (see §3.3.3), the analysis of model (3.1) indicates the parameter region satisfies the condition for case II (see §3.2.1). That is, for any initial pathogen exposure (however small) there will be a positive response. In order for model (3.1) to describe dose-dependent responses, *β* should be larger than *r*. Assuming that *r* in §3.3.3 is not overestimated, this finding shows that the value of *β* obtained from this study could be an underestimate. Otherwise, it indicates that in addition to macrophages other immune cells or chemicals may cause bacterial death or suppress bacterial numbers in the small intestine.

#### 3.3.6. Further estimation of *β* and *α*

Because of the aforementioned limitations concerning *in vitro* human and *in vivo* murine data to inform *β* and *α*, we turn to outbreak scenarios associated to human listeriosis cases. In particular, we make use of our threshold approximation, equation (3.5) (coming from §3.2.4), in conjunction with the Finland butter outbreak surveillance data [18] to estimate *α* and *β* by using a least-squares error minimization.

It was estimated that the mean infection probability and mean dose in that outbreak was 1.0×10^−3^ and 8.2×10^3^ CFU, respectively [18]. While more elaborate fitting schemes involving maximum-likelihood [61] estimates could be used to approximate ranges for *β* and *α*, to illustrate our model approach and minimize extensive computations, we use a least-squares minimization to fit model (3.1) outputs to the butter outbreak data [18]. More precisely, using Matlab's `fmincon' subroutine we minimize the following error function:

3.7 $$E=\sqrt{\sum_{i=1}^{n}{\left(x(d_{i})-\bar{x}(d_{i})\right)}^{2}},\;i=1\ldots n,$$

where *x*(*d*_*i*_) is the probability of infection from surveillance data at dose *d*_*i*_ and *x*(*d*_*i*_) is the model generated probability at dose *d*_*i*_; *n* is the number of observations. Note that *x* is obtained by randomly selecting values of parameters *r*,*α*,*K*,*α* and *β* from their respective distributions (assumed to be normal in this case) and calculating the resulting probability of a positive response from model (3.1) (see §3.2.1). The mean and standard deviation of the first three parameters are estimated in §3.3. The mean and standard deviation of *β* and *α* are estimated from this minimization search (3.7). In particular, the value of *α* is estimated in log scale, that is, we estimate *α*_1_, where *α*=10^*α*_1_^. Since the outbreak information is summarized via the mean infection probability relative to the mean dose, equation (3.7) becomes

3.8 $$E=|x(d_{1})-\bar{x}(d_{1})|,$$

where *d*_1_=8.2×10^3^. The result of this fit (expressed as mean ± standard deviation) indicates that *α*=10^−3.352±0.530^ and *β*=4.904±0.750. With regard to these ranges for *α* and *β*, we mention three key points. (i) The estimated values regarding *β* and *α* depend on the accuracy of the threshold approximation, equation (3.5). We employed the following logic to determine these fits: first, we assumed that formula (3.5) is accurate and proceeded with the minimization calculation, which in turn provided possible ranges for *β* and *α*. We then performed multiple simulations to compare the dose–response function coming from both the long-time solution prediction of model (3.1) and the threshold approximation. A typical result is illustrated in figure 4, showcasing the accuracy of the threshold approximation. (ii) Recall that model (3.1) output quantifies the level of colonization of *L. monocytogenes* in the small intestine. However, the mean infection probability and mean dose from the butter outbreak are built from infection data from [18] and, therefore, our model output *x*(*d*_1_) and the datum *x*(*d*_1_) for the fit are not necessarily in the same category of information. (iii) The outbreak data used to determine *β* and *α* are associated mostly with haematological and organ transplant patients and, therefore, are not representative of a ‘general’ population; see §6 for a discussion concerning the implications of both (ii) and (iii) above.

This completes the estimation of the parameters of model (3.1). To compare our estimation and model generated dose–response curve, we defined the widely used exponential dose–response model

3.9 $$p(d)\;=\;1\;-\;e^{\;-\;R\;\times \;d},\;$$

where *R* is the probability of host–pathogen interaction and *p* is the probability of infection when a given dose *d* is ingested by the host. For comparison, we estimate *R*=1.22×10^−7^ from the same data source [18]. The dose–response curve generated by model (3.4) is also known as the exponential dose–response which has been extensively used by FAO/WHO for microbial risk assessment [18]. Upon completing a sensitivity analysis in the next section, we will vary the most sensitive parameters and compare the predicted dose–response curves with the exponential dose–response (3.4) in §5. The estimated parameters are given in table 2.

## 4. Sensitivity analysis

We estimated the model parameters from the literature and from data fits so that these parameters are closely related to the dynamics of *L. monocytogenes* in the human gut environment. However, since the parameters are from multiple sources and are translated from *in vitro* experiments, uncertainty remains. The following sensitivity analysis reflects the effect of uncertainty on the results of the model as well as identifies which parameters have key roles and which ones dominate model (3.1) prediction. In order to observe the sensitivity of the model solution and threshold on the baseline parameters, we calculate the partial rank correlation coefficient (PRCC) using Latin hypercube sampling (here the parameter values are randomly sampled without replacement from normal distributions across their respective ranges) [62].

### 4.1. Sensitivity of long-time solutions of model (3.1)

**Figure 5.**
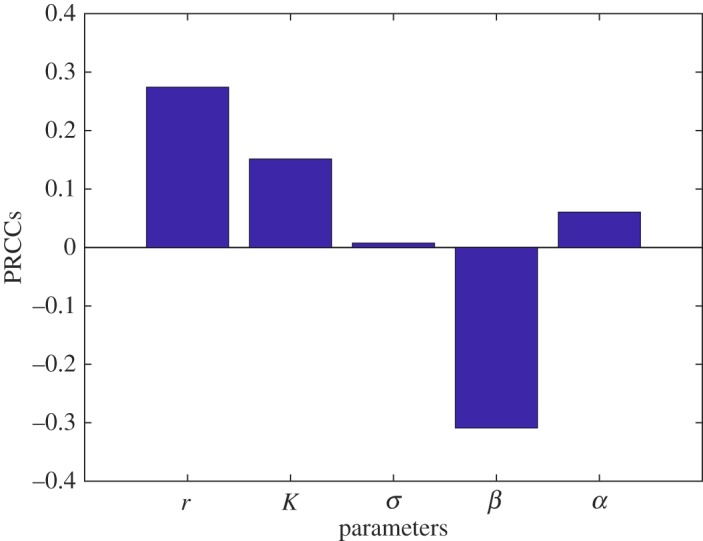
Sensitivity of the model solution on the parameters at dose 2log_10_. It indicates that the model solution is most sensitive to the bacterial growth rate *r* followed by the pathogen killing rate *β* at the low dose.

**Figure 6.**
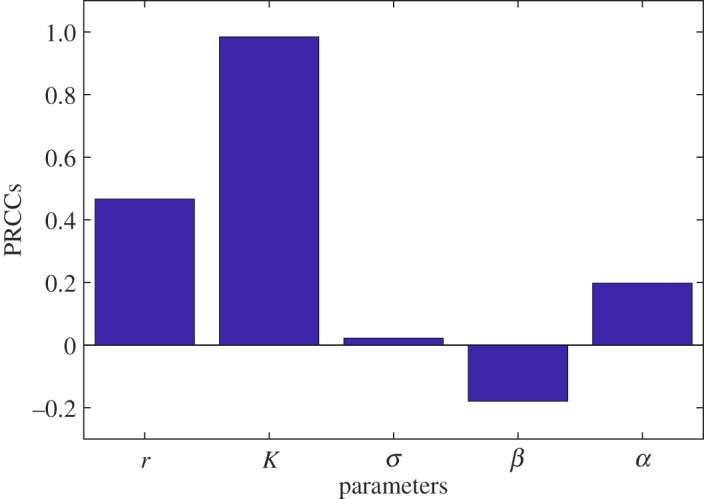
Sensitivity of the model solution on the parameters at dose 8log_10_. It indicates that the model solution is most sensitive to the carrying capacity *K* followed by the bacterial growth rate *r* at the higher dose.

First, we look into the sensitivity of the long-time model solutions. Given an initial value (dose) of the model, PRCCs are calculated over the simulation period. Taking the average of these PRCCs, we obtain the net effect on the model solution from the parameters as illustrated in figures 5 and 6. These show that the model solutions are mostly sensitive to *r*, *σ* and *β* at lower initial doses (2 log_10_ CFU) and to *r*, *K* and *β* at higher initial doses (8 log_10_ CFU). The two parameters *r* and *β* are significant in both scenarios. Given the model form in (3.1), this is a logical result as the pathogen growth rate *r* and the pathogen killing rate (via immune cells, etc.) *β* are parameters, regardless of the initial ingested dose, that can respectively increase/decrease the population level of *L. monocytogenes* in the small intestine. Furthermore, the increased effect of *β* at low doses (figure 5) compared with high doses (figure 6) corresponds to the intuitive notion that the immune response has greater efficacy at lower doses. On the other hand, the role of the carrying capacity *K* is critical when the initial dose is large. This is expected as with a lower dose the pathogen dies out in most cases (any choice of parameters) and could not reach the carrying capacity, while considering a higher dose, the bacteria can quickly reach the carrying capacity and remain bounded by *K*.

### 4.2. Sensitivity of the threshold approximation (3.5)

**Figure 7.**
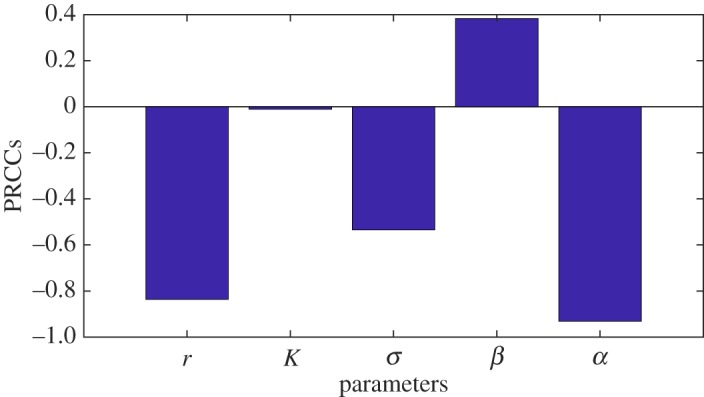
Sensitivity of the threshold (−mL_*_) or the reciprocal of infection probability on the parameters. It shows that the threshold (−mL_*_) is most sensitive to *α* and *r* followed by *σ* and *β*. If *β* decreases or the other parameters increase then the probability of infection will increase according to the relative magnitudes indicated by the PRCCs.

We examine the sensitivity of the threshold (−mL_*_) relative to the model parameters and find that *α* and *r* are the most sensitive parameters as shown in figure 7. Note that the sensitivity of the threshold is inversely related to the sensitivity of the infection probability. That is, if the threshold decreases then the responses or probability of infection increases. In particular, figure 7 indicates that as *r* or *α* increases the threshold decreases which in turn increases the positive responses. Therefore, *α* and *r* are the most critical parameters that dictate the shape of the dose–response curve. The role and significance of *r* on the pathogen survival were previously discussed. The parameter *α* could be associated with the virulence/strain of *L. monocytogenes*. A larger *α* corresponds to a more virulent pathogen that has the potential to avoid the host defence. Thus, a larger *α* should increase the infection probability as seen in figure 7. It is important to mention that if we assume that the parameters are distributed across their respective ranges according to a uniform distribution, the sensitivity of the threshold (3.5) is similar to that of figure 7 with *α* and *r* again possessing the most crucial influence (results not shown).

Finally, it is interesting to note that even though the model solutions are not sensitive to *σ* (figures 5 and 6), the threshold −mL_*_ is (figure 7). Recall that *σ* quantifies the average rate of entry of *L. monocytogenes* into the small intestine. This result indicates that cumulative doses could play an important role for causing infection and increase the infection probability. The parameter *δ* associated with bacteria killing in the stomach has an effect similar to *σ* on the threshold but is not shown in the sensitivity analysis. However, the role and significance of *δ* will be discussed in §6.

## 5. Dose–response curves and comparative results

**Figure 8.**
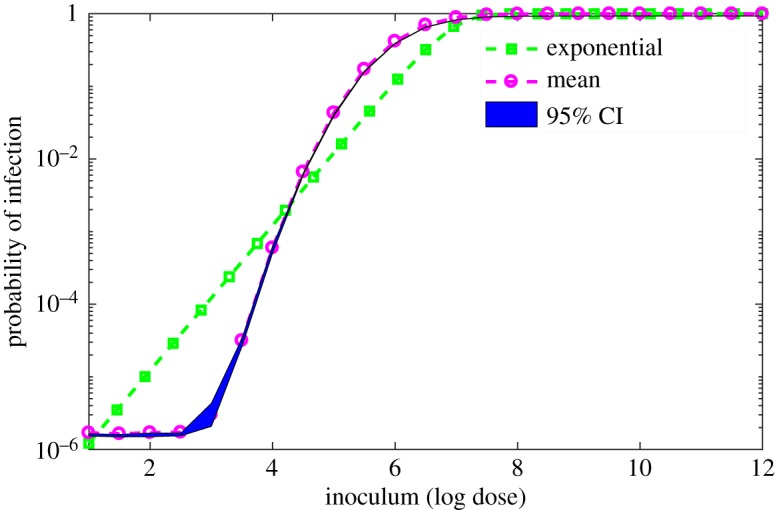
Comparative dose–responses. The exponential dose–response curve is represented by the squares (green) and model (3.1) predicted 95% CI [63] together with the mean curve are given by the shading and circles, respectively.

Using the parameters given in table 2, we use model (3.1) to compute dose–response curves in order to compare our results with the exponential dose–response (Finland butter outbreak [18]). The dose–response function is generated by the model using random samples of *α*, *β*, *r*, *σ* and *K* from the normal distributions across their respective ranges. The predicted dose–response relationship along with the exponential dose–response is shown in figure 8. The significance of our model prediction is that variations in individual immune response across the subpopulation (we are considering the group of haematological and organ transplant patients involved in the Finland outbreak) play a dominant role in dictating the shape of the predicted dose–response relationship. Note that model (3.1) is run with randomly selected parameters so one cannot expect an identical dose–response curve from the repetition of the process. Therefore, in relation to the respective doses *d*_*i*_, we run model (3.1) to produce *n* curves and then compute the 95% CI. Figure 8 illustrates the model predicted dose–response relationship as a shaded region.

For sufficiently high doses, it is reasonable to expect that the variation with regards to the host's environment and ingested pathogen does not significantly alter the response threshold for model (3.1) and hence both the exponential model and model (3.1) should generate similar predictions (figure 8). However, for a lower range of doses, this variation in strain virulence and host immune status considerably affects the response threshold of model (3.1), leading to predictive possibilities outside the scope of the exponential model. One reason for this concerns the fact that the exponential model, used to produce the exponential dose–response, ‘has the oversimplifying assumption of a constant probability of infection following ingestion of *L. monocytogenes* in a given population’ [17]. By contrast, the dose threshold that determines the positive or negative response of model (3.1) is given by a combination of parameters that can vary across strain and individual host characteristics. Compared to the exponential dose–response, figure 8 illustrates that our model predicts lower responses over the low doses (approx. dose 4log_10_ to 1log_10_), but the responses for the high doses (beyond dose 7log_10_) are not significantly different from those of the exponential dose–response (figure 8). Recall that dose–response prediction of model (3.1) represents intestinal colonization rather than infection. In line with this notion, for doses between 1log_10_ and 4log_10_, the results in figure 8 suggest that the exponential dose–response overestimates the infection risk; see §6 for further discussion.

**Figure 9.**
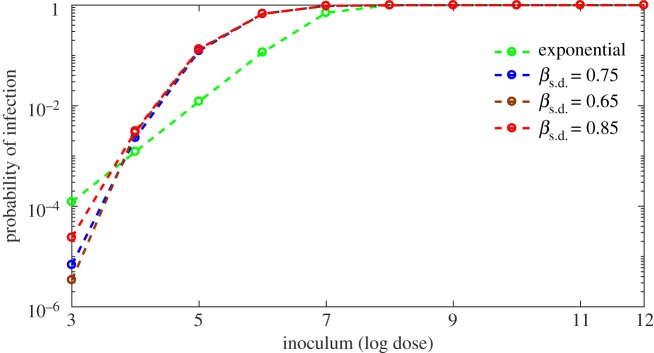
Effect of distribution of *β* on dose–response curve. Exponential (green) curve is generated by (3.9) and three other curves are generated by model (3.1) with the variation of distribution of *β*. Variation of standard deviation (s.d.) does not affect the curves significantly. The estimated standard deviation (s.d.) of *β* is *β*_s.d._=0.75; s.d. is varied by 0.1 to observe the effect of it on the dose–response curve.

**Figure 10.**
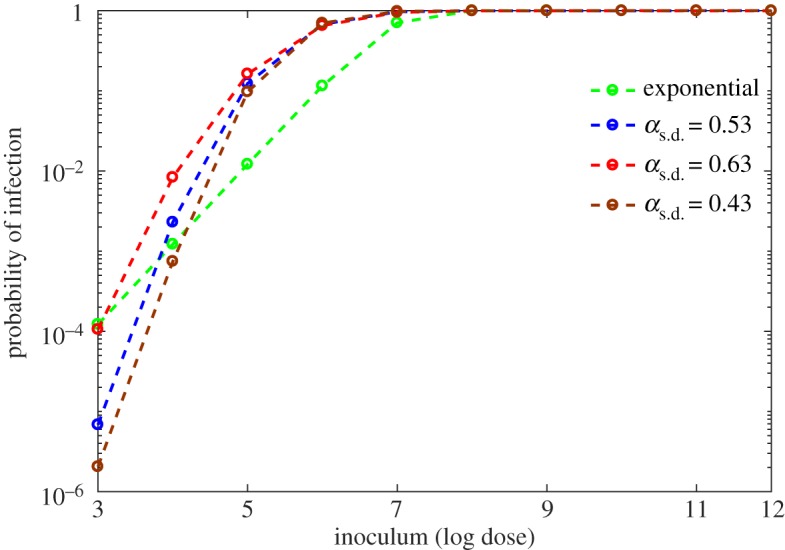
Effect of distribution of *α* on dose–response curve. Exponential (green) curve is generated by (3.9) and three other curves are generated by model (3.1) with the variation of distribution of *α*. Three model generated curves corresponding to different standard deviations are remarkably different. The estimated standard deviation (s.d.) of *α* is *α*_s.d._=0.53; s.d. is varied by 0.1 to observe the effect of it on the dose–response curve.

Since the variation in the host's immune system parameters, *α* and *β*, has a significant influence on the dose–response curve, we investigate the effect on the dose–responses relative to adjusting the standard deviation (s.d.) of the normal distribution for each respective parameter. Figure 9 indicates that the dose–response curve is not sensitive to variations in the spread corresponding to *β*, whereas, with respect to *α*, variation in the spread alters dose–responses considerably (figure 10). As the s.d. of *α* decreases, the response curve begins to decline sharply, indicating less influence on response. However, for a larger s.d. (0.63), the curve shows higher responses through the low doses. This behaviour is consistent with our sensitivity analysis in §4, reinforcing the importance of a quantified connection between the probability of infection in a population and the variation in individual immune responses. Furthermore, the results in figure 10 suggest that the particular distribution of *α* across a population is crucial for dose–response prediction.

## 6. Discussion

Incorporating a mechanistic approach for dose–response modelling is critically important in food safety and microbial risk assessment. Supported by the quantification of key physiological processes, it has an inherent power to connect dose and risk in terms of these mechanisms. In particular, by quantifying the interaction between the human gut environment and *L. monocytogenes*, model (3.1) predicts when the bacteria can cause an infection or at least establish colonization of the small intestinal epithelium. The importance here is that the probability of infection (colonization) is linked to biologically meaningful parameters associated to the particulars of pathogen strain, the digestion process, as well as the human host immune status.

In terms of the recent approaches for characterizing risk and dose–response regarding foodborne pathogens, our mechanistic model serves a complementary role. For instance, the key events framework is a method for studying foodborne disease from the perspective that the individual ‘steps from ingestion to infection are described as a conceptual structure for advancing the dose response models’ [20]. However, characterizing, in general, the cascade of transfer probabilities from one step to the next is non-trivial. While these probabilities may be estimated by using data from animal models or from human outbreaks, such correlative techniques have limited applicability and may not be useful or reliable for predictions under different circumstances. On the other hand, mechanistic models such as model (3.1) are useful as predictive tools to quantify transfer probabilities at potentially any number of steps involved in the key events framework. For example, by interpreting a positive response (given a particular dose) from our model (3.1) as the probability that a certain dose of *L. monocytogenes* can colonize the small intestine, model (3.1) can treat the steps from ingestion to colonization across a range of pathogen strains and characteristics associated to individual human digestive and immune processes within a population.

In this regard, model (3.1) is a powerful tool for predicting risk; however, care must be taken as the underlying assumption that colonization implies infection is not necessarily true (2–10% of the general population may temporarily carry *L. monocytogenes* in their intestinal tract without any negative health effect) [27]. That is, our model outputs for the dose–response really characterize the risk of intestinal colonization. While the distinction between infection and colonization may be quite difficult to describe quantitatively, at the very least the risk of colonization provides an upper bound on the risk of infection. Therefore, in the context of the Finland outbreak, comparing to the exponential model which is commonly used by public health risk assessors, the dose–response relationship predicted by model (3.1) is essentially different for the lower doses (1log_10_ to 4log_10_). However, because the exponential dose–response curve was built from outbreak data involving individuals with compromised health [18], we would expect the difference between the infection and colonization risk (and hence the difference in the predictions from the respective models) to be smaller than the difference involving a more general or heterogeneous population. To appreciate this, note that the exponential dose–response curve is given by *p*(*d*)=1−e^−*R*×*d*^, where *R*=1.22×10^−7^ as compared to an *R* value of 1.19×10^−10^ corresponding to a more general population [17]. Also, in the context of a *L. monocytogenes* outbreak among haematological and organ transplant patients, the high risk of infection (as compared with a general human population) provides some justification for using the Finland outbreak data to determine the ranges for the immune parameters *α* and *β*. That is, while we indicated that *x*(*d*) and *x*(*d*) (coming from formula (3.8)) are not exactly in the same category of information, that is, *x*(*d*) quantifies probability of infection from the outbreak data and *x*(*d*) represents that colonization probability predicted from model (3.1), this discrepancy is much less than in the context of a general population. Furthermore, it is important to mention that many studies that use animal surrogates to determine dose–response outcomes associated to *L. monocytogenes* do not clearly articulate the distinction between colonization and infection [10,11,13].

In line with predicting the risk of infection or at least colonization in the small intestine, the utility of model (3.1) relies on the determination of appropriate parameter distributions. Certain parameters, such as the growth rate, *r*, of a particular strain of *L. monocytogenes* attempting to colonize the small intestine, are impossible to measure directly in humans. Nevertheless, because the parameters in model (3.1) provide a biological connection with the mathematical forms used to represent the underlying mechanisms, as opposed to merely descriptive values that may lack clear biological meaning, they can be estimated with data from a variety of experiments rather than relying only on animal models or past outbreak scenarios.

Dictating the dose–response relationship, the sensitivity analysis in §4 indicates that *α* is the most influential parameter followed by the growth rate *r* (figure 7). In fact, *α* is the key parameter for model (3.1) in that it mathematically allows the possibility of dose-dependent relationships. In other words, when *α*=0, the model outcomes are no longer dependent on the initial doses (see case III in §3.2.4). Therefore, proper interpretation and appropriate values of *α* are necessary. In addition to determining the correct range for *α*, our analysis in §4 illustrates that the specific distribution of *α* on its range significantly affects the dose–response prediction of model (3.1). Recall that *α* quantifies the ability of the pathogen in question to reduce the efficacy of the immune system killing rate, *β*, as the pathogen multiplies. While it may be difficult to characterize the distribution of *α* from an appropriate *in vitro* experiment tracking the listericidal activity of human small intestine macrophages, the mean and standard deviation associated to *α* may suffice. That is, given the mean and standard deviation of *α*, one can input a variety of distributions into model (3.1), resulting in a thickened curve or ‘band’ of positive response versus ingested dose that elucidates best/worst case possibilities. Note that this type of analysis can be administered for other significant parameter values, quantifying the sensitivity of the dose–response function relative to the distribution of these parameters. This work is relegated to future research as specific data are needed to help ensure at least partial identifiability of such parameters. See the next paragraph for suggestions regarding such data collection.

In addition, *β* is a critically important parameter as it is closely related to *α* in the host–pathogen interaction. Owing to the lack of human data in the literature regarding the interaction between human small intestine macrophages and *L. monocytogenes*, we back calculated ranges for *β* and *α* using the surveillance data from the Finland butter outbreak [18]. However, these results depend on the fact that the underlying population is unhealthy and that at relatively higher doses, both the exponential model and model (3.1) should predict similar positive responses, as discussed in §3.3.6. In the light of the aforementioned suppositions, simplifying assumptions used to build the exponential curve, and the fact that the response threshold (−mL_*_) for model (3.1) is highly sensitive to *α*, the ranges of *α* and *β* should ideally be tested against estimations from a single study that incorporates different initial inoculum levels of *L. monocytogenes*. In particular, we suggest that scientific experiments to estimate these parameters involve a simulated human gut environment. Lately, the human gastrointestinal microbiome has been studied using a new *in vitro* model known as the HIM module [64]. As this model serves to mimic the fundamental *in vivo* mechanisms that govern the gastrointestinal microbial community, it may also prove to be an insightful parametrization tool for new mechanistic dose–response models such as model (3.1). Classifying distributions of *α* and *β* in this context by studying intestinal antimicrobial (immune) cells from various immunocompromised subgroups may prove to be invaluable.

From a mathematical perspective, model (3.1) exhibits bistable phenomena due to the equation for *L*_*i*_, describing the evolution of the pathogen in the environment of the small intestine. The first part of this equation describes the growth of the bacteria and the second part

6.1 $$D(L_{i})=\frac{\beta L_{i}}{1+\alpha L_{i}}$$

describes the killing of bacteria. This killing form is important as it determines the bistable phenomena. Any mathematical form that can exhibit bistability and is biologically feasible could be a candidate to replace *D*(*L*_*i*_). A possible candidate for *D*(*L*_*i*_) is given by

6.2 $$D(L_{i})\;=\;\beta \;e^{\;-\;\alpha L_{i}}L_{i}.$$

We aim to investigate, in upcoming projects, which mathematical form could best fit surveillance/ experimental data and interpret the host–pathogen interaction.

On another front, the parameter *δ*, which quantifies the pathogen killing rate due to the pH level in the stomach, is crucial for determining dose–responses. In this study, we fixed *δ* at its mean value estimated from the literature (see §3.3.1), and therefore it was not included in the sensitivity analysis in §4. However, because of the potential variation in the stomach environment, due to the use of H_2_ blockers, or to differences in the ingested food matrix that serves as a vehicle of contamination, as well as pathogen strain and history, it may be important to quantify the dynamics of *δ* in terms of these factors. At the very least, classifying an average value of *δ* as a function of initial dose and food type would enable model (3.1) to become a profitable tool for regulatory agencies, especially in the light of the fact that non-traditional foods including ‘pre-cut fruits and vegetables, ice-cream, cantaloupe, mung bean sprouts, stone fruits and caramel apples’ have recently been associated with listeriosis [27].

In the quest of understanding the dose–response relationship of *L. monocytogenes* in the human host, testing novel approaches is of great priority. Mechanistic models could be the potential tools to elucidate further insights of this paradigm. Independent studies supported by scientific experiments are necessary to validate this novel approach.

## Data accessibility

The dataset associated with figure 8 is available at [63]. All the graphs are generated using Matlab software which is obtained from https://www.mathworks.com/products/matlab.html. The Matlab code is also available in [63].
